# Ether with Cod Liver Oil

**Published:** 1879-06

**Authors:** 


### ETHER WITH COD LIVER OIL.

The fact that cod liver oil can not be tolerated in a very large number of cases where the use of the remedy is indicated, led the New York Therapeutical Society to refer to a committee, for investigation, the claims of Dr. Foster, who first suggested the combination of the oil with ether as a means of overcoming the difficulty. The committee, after an examination of ninety-four cases, report that the evidence before them warrants the conclusions: (1) That the addition of ether to cod liver oil, in about the proportion of fifteen minims to each half ounce (or an equivalent amount of Hoffmann's anodyne instead of ether), will succeed in the vast majority of cases in enabling the patient to take the oil, even though it previously disagreed; (2) that in some cases in which the oil still disagrees after the addition of the ether, the difficulty may be overcome by giving the ether separately from fifteen minutes to half an hour after the oil is taken. No facts were laid before the committee from which they could judge as to whether the etherized oil is superior to the plain oil in its ultimate effect upon nutrition, supposing them to be equally well tolerated by the stomach —*Scientific American.*

